# Learning Trajectory Distributions for Assisted Teleoperation and Path Planning

**DOI:** 10.3389/frobt.2019.00089

**Published:** 2019-09-24

**Authors:** Marco Ewerton, Oleg Arenz, Guilherme Maeda, Dorothea Koert, Zlatko Kolev, Masaki Takahashi, Jan Peters

**Affiliations:** ^1^Intelligent Autonomous Systems Group, Department of Computer Science, Technische Universität Darmstadt, Darmstadt, Germany; ^2^Perception and Activity Understanding Group/Robot Learning and Interaction Group, Idiap Research Institute, Martigny, Switzerland; ^3^ATR Computational Neuroscience Laboratory, Department of Brain Robot Interface, Kyoto, Japan; ^4^Preferred Networks, Inc., Tokyo, Japan; ^5^Takahashi Laboratory, Department of System Design Engineering, Faculty of Science and Technology, Keio University, Yokohama, Japan; ^6^Robot Learning Group, Max Planck Institute for Intelligent Systems, Tübingen, Germany

**Keywords:** assisted teleoperation, path planning, movement primitives, reinforcement learning, policy search, Gaussian processes

## Abstract

Several approaches have been proposed to assist humans in co-manipulation and teleoperation tasks given demonstrated trajectories. However, these approaches are not applicable when the demonstrations are suboptimal or when the generalization capabilities of the learned models cannot cope with the changes in the environment. Nevertheless, in real co-manipulation and teleoperation tasks, the original demonstrations will often be suboptimal and a learning system must be able to cope with new situations. This paper presents a reinforcement learning algorithm that can be applied to such problems. The proposed algorithm is initialized with a probability distribution of demonstrated trajectories and is based on the concept of relevance functions. We show in this paper how the relevance of trajectory parameters to optimization objectives is connected with the concept of Pearson correlation. First, we demonstrate the efficacy of our algorithm by addressing the assisted teleoperation of an object in a static virtual environment. Afterward, we extend this algorithm to deal with dynamic environments by utilizing Gaussian Process regression. The full framework is applied to make a point particle and a 7-DoF robot arm autonomously adapt their movements to changes in the environment as well as to assist the teleoperation of a 7-DoF robot arm in a dynamic environment.

## 1. Introduction

Learning from demonstrations is a promising approach toward human-robot co-manipulation and teleoperation. With this approach, a user can easily demonstrate trajectories to a robot, for instance in gravity compensation mode. Subsequently, the robot fits a model to these trajectories, which allows it to assist the user in the execution of repetitive tasks. The robot assistance can potentially reduce the cognitive load in the user and prevent unintended collisions. Moreover, training a teleoperated robot through demonstrations can give it a certain degree of autonomy, which is desirable in the face of communication latency and intermittency.

Recently, methods have been proposed to assist humans in co-manipulation and teleoperation tasks given demonstrated trajectories (Raiola et al., 2015; Havoutis and Calinon, 2016, 2017). Our work contributes to this field by providing a new reinforcement learning algorithm, **P**earson-Correlation-Based **R**elevance Weighted Policy **O**ptimization (PRO), to improve upon demonstrated trajectories when these are suboptimal or when solutions to new situations must be found. These trajectories need to be optimized with respect to objectives, such as minimizing distances to via points, keeping a certain minimum distance from obstacles, achieving minimal length, minimal jerk, etc.

**Figure 1:**
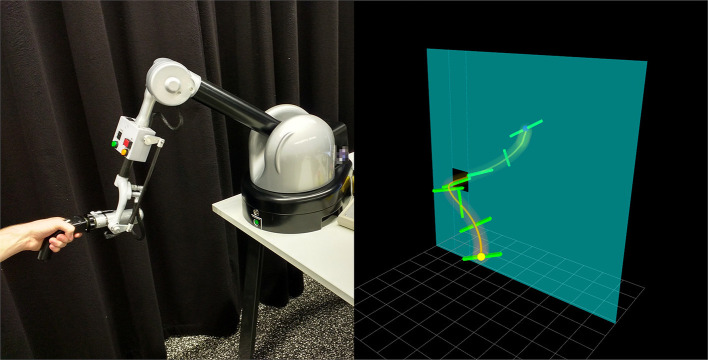
A haptic device, the Haption Virtuose 6D, is operated by a user to move a beam in a virtual environment. The haptic device uses a trajectory distribution learned with Pearson-Correlation-Based Relevance Weighted Policy Optimization (PRO) to assist the user in moving the beam from a start position and orientation to an end position and orientation through a window in the wall.

In Ewerton et al. (2018), we have introduced the concept of relevance to optimize trajectory distributions. By using this concept, it is possible to preserve the variance of trajectory parameters that are not relevant to the objective currently being optimized for. This property is helpful for optimizing trajectories sequentially with respect to multiple objectives because the search for optimal parameters for a certain objective does not disturb other parameters that are irrelevant to the objective currently under consideration. In that work, a relevance function was represented by a weighted sum of basis functions. The relevance weights were learned through an iterative process. The new algorithm presented in this paper, PRO, is based on the insight that the Pearson correlation coefficient (Benesty et al., 2009) can be used to determine how each trajectory parameter influences each objective. It does not require designing basis functions for the relevance. Moreover, the relevance is now determined in one shot in contrast to the previous iterative approach. The efficacy of the proposed algorithm is demonstrated in an assisted teleoperation experiment involving a haptic device, the Haption Virtuose 6D (see Figure 1).

**Figure 2:**
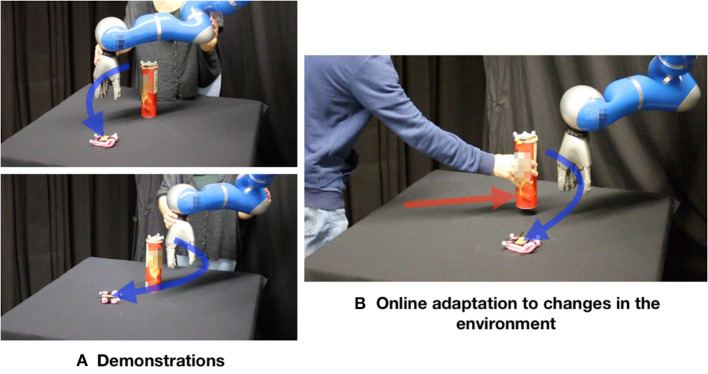
In this experiment, the robot has to reach the target (pink object) while avoiding the obstacle (cylinder). **(A)** Demonstrations. **(B)** Our learning system is able to adapt on the fly a distribution of trajectories to changes in the environment. This distribution can be used for path planning tasks as well as for co-manipulation and assisted teleoperation in dynamic environments.

Finally, we extend PRO with Gaussian Processes (GP) regression to cope with dynamic environments. A GP is initialized with demonstrations and prior knowledge. Given a new environment, it outputs a distribution of trajectories which guides the movements of a robot to solve a certain task. PRO is used to optimize upon the GP inferences, gradually improving the mapping from environment to trajectory distribution. After a phase of self-optimization, our learning system is able to compute successful trajectories on the fly face to changes in the environment. This paper presents applications of this framework in two problems. The first problem involves a point particle that needs to achieve a dynamic target while avoiding moving walls. The second one consists in controlling a 7-DoF robot arm to reach a target while avoiding a cylinder on a table. Both the position of the target and the position of the cylinder can be changed by a human (see Figure 2).

The main contribution of this paper is a new algorithm that learns trajectory distributions to solve planning tasks both in static and dynamic environments. The learned trajectory distributions are appealing because they function as virtual guides for users in shared-autonomy tasks, e.g., assisted teleoperation, as it is demonstrated in our experiments. Excerpts of this work have been accepted for presentation at Ewerton et al. (2019).

## 2. Related Work

Assisted teleoperation is intensely investigated because, at the same time that it is useful to leverage the superior cognitive capabilities of a human operator, humans may not have the necessary accuracy or perception of the environment to solve a task. In addition, teleoperation involves several technical challenges, such as dealing with communication delays and noise. Xu et al. (2016) presented a framework to assist users in teleoperation tasks with visual and haptic feedback based on artificial potential fields and on the parameters of geometric and dynamic models. The parameters of the dynamic models are online updated. That framework helps the user to succeed in teleoperation tasks even in the presence of round-trip communication delays of 2 s. In our work, we address the problem of optimizing suboptimal trajectories and use the solution of this optimization problem to help the user to perform a teleoperation task with less effort. The framework proposed in Xu et al. (2016) and our approach could be used together to address problems, such as delay while at the same time guiding the user to perform more efficient trajectories than he/she could originally perform.

To assist users in teleoperation or potentially in co-manipulation tasks given suboptimal attempts or demonstrations, our algorithm has to be able to refine continuous paths to avoid obstacles, pass through via points, etc. CHOMP (Ratliff et al., 2009) and STOMP (Kalakrishnan et al., 2011) are two prominent methods for continuous path refinement. CHOMP is a gradient-based optimization technique to minimize a cost function while changing an initial trajectory as little as possible. STOMP is a gradient-free, stochastic trajectory optimization technique based on Policy Improvement with Path Integrals (PI^2^) (Theodorou et al., 2010). Our algorithm, PRO, presents some similarities to STOMP. It is a gradient-free, stochastic trajectory optimization technique based on Reward Weighted Regression (RWR) (Peters and Schaal, 2007). While in STOMP, trajectories are generated by perturbing an initial trajectory with a certain noise, in our work, a distribution of trajectories based on demonstrations (and potentially also on prior knowledge) is optimized. Differently from the original works on CHOMP and STOMP, in our work, a mapping from environment configurations to probability distributions is learned. Subsequently, this mapping is used to adapt movements on the fly to changes in the environment, without the need for further optimization. The query of the appropriate trajectory distribution given the environment configuration is made in one shot while optimizations, such as the ones performed by CHOMP and STOMP are iterative procedures. Therefore, our framework can achieve online adaptation without making so much computation online. Moreover, computation is saved by using the solutions to similar environments to infer the solution to a new environment.

An effective method for assisting users in shared-autonomy tasks, e.g., co-manipulation, with probabilistic models learned from demonstrations has been proposed in Raiola et al. (2015). In that paper, Gaussian Mixture Models (Calinon et al., 2007) are used to create multiple probabilistic virtual guides, which constrain the movements of the user to a region close to the demonstrations.

As in our work, in Havoutis and Calinon (2016), probabilistic models are used to assist users in teleoperation tasks with shared control. In that work, task-parameterized Gaussian Mixture Models (TP-GMMs) (Calinon, 2016) have been used to encode the probability distribution of demonstrated trajectories. Gaussian Mixture Regression (GMR) (Calinon, 2016) has been used to generate a behavior according to the learned model. The learning agent assists the user with the teleoperation of a device to scan a surface.

Our work is in line with Raiola et al. (2015) and Havoutis and Calinon (2016), with the important difference that our approach addresses cases where demonstrations are suboptimal or when the learned model cannot generalize well enough to a new scenario. This is possible due to the optimization of the original distribution through reinforcement learning. An approach for improving upon suboptimal initial demonstrations is presented in Abi-Farraj et al. (2017). Nevertheless, that approach is based on iterative refinement by the human user instead of reinforcement learning.

Learning from demonstrations has also been applied in supervisory control. In this paradigm, after training, the remote system can execute a task autonomously, needing only high-level task goals to be provided by the user. In Havoutis and Calinon (2017), task-parameterized hidden semi-Markov models (TP-HSMMs) are used to build probabilistic models of manipulation motions and Model Predictive Control (MPC) is used to execute these motions. In that work, TP-HSMMs have been shown to generalize better to changes in the environment than Probabilistic Movement Primitives (ProMPs) (Paraschos et al., 2018), which are used in our work. We believe that our work can contribute to enhancing the generalization capabilities of frameworks using probabilistic models, such as TP-HSMM and ProMP by using reinforcement learning to let the remote system look for solutions to new tasks by trial and error.

Previous works have proposed approaches to adapt initial movements learned from demonstrations to new environments with obstacles (Koert et al., 2016; Osa et al., 2017; Rana et al., 2018). As a contribution to this area of research, our approach creates in an offline self-optimization procedure a mapping between environment configurations and trajectory distributions, which can be used to online adapt these distributions in dynamic environments. This is achieved by using Gaussian Process (GP) regression to map variables describing the environment to parameters (mean vector and covariance matrix) of a probability distribution of trajectories in the form of a ProMP. Our proposed reinforcement learning algorithm, Pearson-Correlation-Based Relevance Weighted Policy Optimization (PRO), is used to iteratively refine this mapping. In a sense, PRO is providing the GPs with new optimized ProMPs for any given environment, which gradually improves the mapping. PRO performs Reinforcement Learning while GP regression performs Supervised Learning. This process resembles the way Guided Policy Search (GPS) (Levine et al., 2016) uses trajectory optimization in combination with the constraint that the actions output by a Convolutional Neural Network (CNN) must track the optimized trajectories. In our approach, PRO assumes the role of the trajectory optimizer while the GPs assumes the role of the CNN. In contrast to GPS, while the CNN outputs actions for any given state, in our approach, GP regression outputs ProMPs (distributions of trajectories) for any given environment. These distributions are used in our work as virtual guides that can constrain the movements of the human and the robot to certain regions of the state space.

## 3. Pearson-Correlation-Based Relevance Weighted Policy Optimization (PRO)

This section explains PRO, a policy search algorithm which uses the relevance of each trajectory parameter to each optimization objective to optimize policies in the form of trajectory distributions. The relevance is computed in one shot by using Pearson correlation coefficients.

### 3.1. Relevance Functions

PRO is a stochastic policy search algorithm based on Reward Weighted Regression (RWR) (Peters and Schaal, 2007). In each iteration of RWR applied to trajectory optimization, trajectory parameters are sampled from a probability distribution, e.g., a Gaussian. Subsequently, the parameters of that probability distribution, e.g., mean vector and covariance matrix, are optimized to maximize the expected reward.

**Figure 3:**
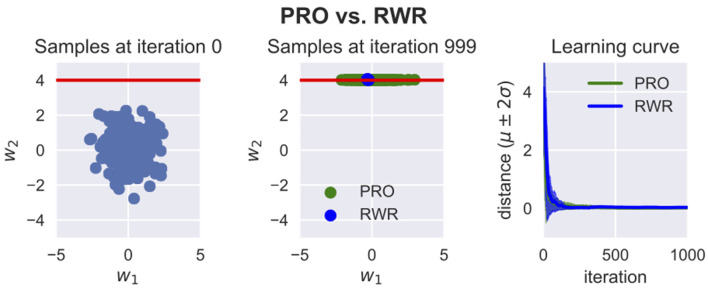
Pearson-Correlation-Based Relevance Weighted Policy Optimization (PRO) vs. Reward Weighted Regression (RWR). Here, *w*_1_ and *w*_2_ are the trajectory parameters. These trajectory parameters could be for example the weights for some basis functions. For visualization purposes, it is assumed in this example that only two parameters suffice to parameterize the trajectories. The red line represents the region in the space of trajectory parameters where the reward is the maximum. The reward for any point in this space is *R* = exp (−*βd*), where *β* is a hyperparameter chosen by the user and *d* is the distance between the point and the red line. Both RWR and PRO were applied to optimize a Gaussian distribution of $w={\left[w_{1},w_{2}\right]}^{⊤}$ with 1,000 iterations and 200 samples per iteration. The variances of RWR collapse while PRO is able to keep the variance of *w*_1_ because this parameter is not relevant to this optimization problem. PRO can thus optimize the mean and variance of a certain parameter without disturbing the mean and variance of parameters which are irrelevant to the objective being optimized for. This property is helpful when sequentially optimizing trajectory distributions with respect to several objectives or to conserve as much as possible of the variance of the initial distribution.

In PRO, the relevance of each trajectory parameter to each optimization objective is estimated. This information is then used to determine how the trajectory parameters should be sampled when optimizing the distribution of trajectory parameters with respect to each objective. This procedure prevents undesirable changes in the distribution of trajectory parameters that do not influence the objective under consideration. On the other hand, only the trajectory parameters that actually influence the objective under consideration are subject to exploration in the sampling procedure. Figure 3 illustrates the difference between RWR and PRO.

The key observation in PRO is that the relevance of the trajectory parameter *w*_*n*_ to the objective *o*, denoted by *f*_*o*_(*n*), can be represented by the absolute value |*ρ*_*n, o*_| of the Pearson correlation coefficient

(1) $$\begin{array}{l} \rho_{n,o}=\frac{\text{cov}\left(w_{n},o\right)}{\sigma_{w_{n}}\sigma_{o}}, \end{array}$$

where cov(*w*_*n*_, *o*) is the covariance between *w*_*n*_ and the value of the objective *o*, σ_*w*_*n*__ is the standard deviation of *w*_*n*_ and σ_*o*_ is the standard deviation of the values of the objective *o*. The values *ρ*_*n, o*_ can be computed from samples of $w={\left[w_{1},\cdots \;,w_{N}\right]}^{⊤}$, where *N* is the number of trajectory parameters. In this work, the samples ***w*** to compute *ρ*_*n, o*_ are drawn from a Gaussian with mean vector ***μ*_*w*_** and covariance matrix $\sigma_{\text{relevance}}^{2}I_{N\times N}$. The mean ***μ*_*w*_** can be based on demonstrations and $\sigma_{\text{relevance}}^{2}$ is chosen by the user to produce small disturbances around the mean. The underlying assumption in this method of quantifying relevance is that the trajectory parameters are locally linearly correlated with the optimization objectives. This is why $\sigma_{\text{relevance}}^{2}$ needs to be small. For each ***w***, the value of each objective *o* is computed. Given the samples ***w*** and the corresponding objective values *o*, the computation of *ρ*_*n,o*_ is straightforward and can be implemented with a single line of code using libraries, such as NumPy.

The Pearson correlation coefficient *ρ*_*X,Y*_ of any two random variables *X* and *Y* is a measure of the linear correlation between *X* and *Y* and −1 ≤ *ρ*_*X,Y*_ ≤ 1. Thus, the relevance function *f* = |*ρ*_*X,Y*_| is such that 0 ≤ *f* ≤ 1. The relevance *f*_*o*_(*n*) of *w*_*n*_ to the objective *o* expresses how strongly changes in *w*_*n*_ are linearly correlated to changes in the values of the objective *o*. In practice, to explore a large range of parameter values, it is helpful to normalize the relevance functions *f*_*o*_(*n*) such that their maximum value is 1 instead of a smaller value. The normalized relevance functions are thus given by $\frac{f_{o}\left(n\right)}{{\text{max}}_{n}f_{o}\left(n\right)}$. Assuming our probability distribution of trajectory parameters *w*_*n*_ is a Gaussian, i.e., $w\sim N\left(\mu_{w},\Sigma_{w}\right)$, PRO samples *w*_*n*_ from the distribution $N\left(\mu_{w},\Sigma_{w}^{f_{o}}\right)$, where

(2) $$\begin{array}{l} \Sigma_{w}^{f_{o}}=\text{diag}\left(\sigma_{w_{1}}^{2}f_{o}\left(1\right),\cdots \;,\sigma_{w_{N}}^{2}f_{o}\left(N\right)\right). \end{array}$$

In our work, the initial distribution $N\left(\mu_{w},\Sigma_{w}\right)$ is based on demonstrations. The parameters ***w*** for each demonstration are learned through Linear Ridge Regression and Maximum Likelihood Estimation (MLE) is used to determine ***μ***_***w***_ and **Σ**_***w***_.

### 3.2. Optimization of Trajectory Distributions Using Relevance Functions

Once a number *S* of trajectory parameter vectors ***w*** have been sampled from $N\left(\mu_{w},\Sigma_{w}^{f_{o}}\right)$, Reward Weighted Regression (RWR) is used to optimize the mean ***μ***_***w***_ and the covariance matrix $\Sigma_{w}^{f_{o}}$ of this distribution to maximize the expected reward. The optimization problem

(3) $$\begin{array}{l} \left\{\mu_{w}^{k+1},C^{k+1}\right\}=\underset{\left\{\mu_{w},{\Sigma_{w}}^{f_{o}}\right\}}{\text{arg max}}\overset{S}{\underset{i=1}{\sum }}R_{o,i}N(w_{i};\mu_{w},\Sigma_{w}^{f_{o}}) \end{array}$$

has the solution

(4) $$\begin{array}{lll} \mu_{w}^{k+1} & = & \frac{\sum_{i=1}^{S}R_{o,i}w_{i}}{\sum_{i=1}^{S}R_{o,i}},\\ C^{k+1} & = & \frac{\sum_{i=1}^{S}R_{o,i}\left(w_{i}-\mu_{w}^{k}\right){\left(w_{i}-\mu_{w}^{k}\right)}^{⊤}}{\sum_{i=1}^{S}R_{o,i}}. \end{array}$$

The variable *k* in the expressions above represents the iterations of the algorithm. The variable *S* is the number of sampled vectors ***w***. This number is chosen by the user of the algorithm to enable an adequate approximation of the expected reward. The variable *R*_*o, i*_ represents the reward with respect to objective *o* obtained by the sampled trajectory *i*. The reward is non-negative and usually has the form *R*_*o, i*_ = exp (−*βo*(*i*)), where *o*(*i*) is the value obtained by the sampled trajectory *i* for objective *o* and *β* is a hyperparameter chosen by the user.

Finally, the new covariance matrix **Σ_*w*_** is determined. It is a diagonal matrix with the variances in the diagonal given by

(5) $$\begin{array}{l} \sigma_{w_{n},k+1}^{2}=\left(1-f_{o}\left(n\right)\right)\sigma_{w_{n},k}^{2}+f_{o}\left(n\right)C_{nn}^{k+1}, \end{array}$$

where $\sigma_{w_{n},k}^{2}$ is the variance of *w*_*n*_ in iteration *k* and $C_{nn}^{k+1}$ is the element at row *n* and column *n* of the covariance matrix ***C***^*k*+1^.

Equation (5) keeps the variance of irrelevant trajectory parameters unchanged and updates the variance of relevant trajectory parameters. If *f*_*o*_(*n*) = 0, for example, $\sigma_{w_{n},k+1}^{2}=\sigma_{w_{n},k}^{2}$, i.e., the variance of *w*_*n*_ at iteration *k* + 1 is the same as at iteration *k*. On the other hand, if *f*_*o*_(*n*) = 1, $\sigma_{w_{n},k+1}^{2}=C_{nn}^{k+1}$, i.e., the variance of *w*_*n*_ at iteration *k* + 1 is the result of the RWR optimization at iteration *k*, yielding $C_{nn}^{k+1}$. For other relevance values, which must lie by definition between 0 and 1, the new variance is a weighted average of its previous value and the optimized one.

**Figure 4:**
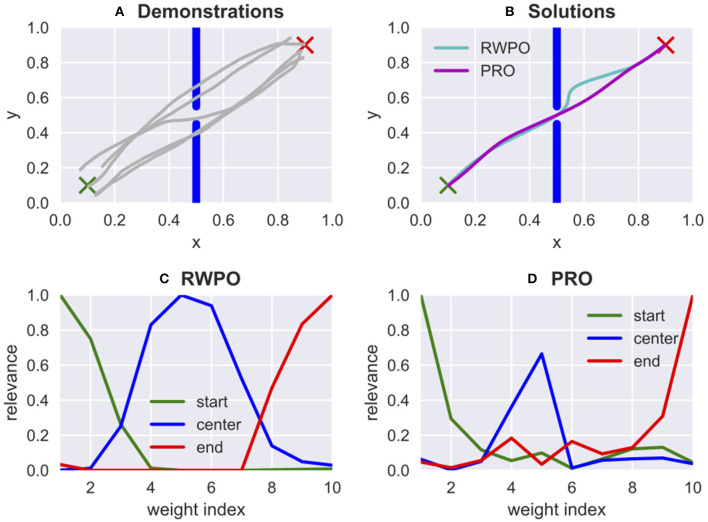
Comparison between Relevance Weighted Policy Optimization (RWPO), proposed in Ewerton et al. (2018), and Pearson-Correlation-Based Relevance Weighted Policy Optimization (PRO), proposed in this paper. In this problem, a trajectory must be found that starts at the green ×, goes through the window in the center and ends at the red ×. RWPO took 84.47 s to learn the relevance functions while PRO took only 0.02 s. Both algorithms were implemented in Python. The machine used for both computations was the same. The hyperparameters for RWPO were the same as used in Ewerton et al. (2018). PRO used 200 trajectory samples to compute the relevance function. The vast difference in execution time is due to the iterative nature of the relevance computation in RWPO while PRO has a one-shot approach to compute the relevance. **(A)** Five demonstrations provided by a user. **(B)** Both algorithms find solutions that satisfy the given criteria. The larger deviation from the direct path observed in the solution by RWPO does not represent an error because there is no cost for larger deviations from the direct path. The cost function used by both algorithms is the same and depends only on the distances to the start, window center and end. **(C,D)** Relevance for the 10 weights that parameterize the *x* trajectory computed by RWPO and by PRO, respectively. The relevance for the 10 weights that parameterize the *y* trajectory has not been plotted here. Differently from RWPO, PRO does not use basis functions for the relevance.

Algorithms 1, 2 present a description of PRO in the form of pseudocode. Figure 4 shows a comparison between the algorithm proposed in Ewerton et al. (2018), Relevance Weighted Policy Optimization (RWPO), and the one proposed in this paper, PRO. PRO has two main advantages over RWPO: (1) in PRO, it is not necessary to design problem-specific basis functions for the relevance; (2) The relevance computation in PRO is performed in one shot, which is much faster than the iterative procedure used in RWPO. A fast computation of the relevance is crucial because, in general, the relevance may need to be reevaluated during the optimization of the trajectory distributions. This necessity is due to the fact that the relevance is computed from samples of a Gaussian with mean ***μ*_*w*_** and covariance matrix $\sigma_{\text{relevance}}^{2}I_{N\times N}$. Therefore, the relevance depends on ***μ*_*w*_**, which needs to be optimized.

**Algorithm 1:** Relevance learning

1:	**Inputs:** mean ***μ*_*w*_**, variance $\sigma_{\text{relevance}}^{2}$ and objective function corresponding to objective *o*
2:	Sample trajectory parameters ***w*** from $N\left(\mu_{w},\sigma_{\text{relevance}}^{2}I_{N\times N}\right)$
3:	**for** each sample ***w*** **do**
4:	Compute the value for the objective *o* obtained by the trajectory with parameters ***w***
5:	**end for**
6:	**for** each *w*_*n*_ **do**
7:	Compute the Pearson correlation coefficient *ρ*_*n, o*_ (Equation1)
8:	Compute the relevance function *f*_*o*_(*n*) = |*ρ*_*n, o*_|
9:	Normalize the relevance function
10:	**end for**
11:	**return** the normalized relevance functions *f*_*o*_(*n*)

**Algorithm 2:** Pearson-Correlation-Based Relevance Weighted Policy Optimization

1:	**Inputs:** mean ***μ*_*w*_**, covariance **Σ_*w*_** of trajectory parameters ***w***, variance $\sigma_{\text{relevance}}^{2}$ and objective functions
2:	**repeat**
3:	**for** each objective *o* **do**
4:	Compute relevance functions *f*_*o*_(*n*) (Algorithm 1)
5:	Compute matrix $\Sigma_{w}^{f_{o}}$ (Equation 2)
6:	Sample trajectory parameters ***w*** from $N\left(\mu_{w},\Sigma_{w}^{f_{o}}\right)$
7:	**for** each sample ***w***_*i*_ **do**
8:	Compute the reward *R*_*o, i*_ of the trajectory with parameters ***w***_*i*_ associated with objective o
9:	**end for**
10:	Update ***μ*_*w*_** and compute ***C*** (Equations 4)
11:	Update the variances of the trajectory parameters $\sigma_{w_{n}}^{2}$ (Equation 5)
12:	**end for**
13:	**until** convergence of the rewards *R*_*o, i*_
14:	**return** the mean ***μ*_*w*_** and the variances $\sigma_{w_{n}}^{2}$

## 4. Online Adaptation of Trajectory Distributions

In this work, Probabilistic Movement Primitives (ProMPs) (Paraschos et al., 2018) are used to represent trajectory distributions. In ProMPs, each trajectory is approximated by a weighted sum of Gaussian basis functions evenly spaced along the time axis. Each trajectory can thus be represented by a vector of weights $w={\left[w_{1},\cdots \;,w_{N}\right]}^{⊤}$, where *N* is the number of Gaussian basis functions. Given a number of demonstrated trajectories for a certain task, a Gaussian distribution $N\left(\mu_{w},\Sigma_{w}\right)$ of ***w*** is computed through Maximum Likelihood Estimation (MLE).

ProMPs allow for computing the posterior probability distribution of trajectories given via points. This operation, however, produces sensible results only if the via points are close to the original ProMP. For our purposes, we need to adapt ProMPs to environment configuration variables like via points and others also when they are very different from the configurations observed during the demonstration phase.

**Figure 5:**
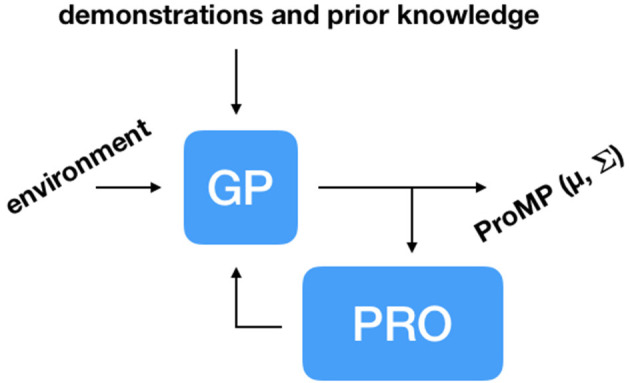
Architecture to adapt trajectory distributions, in this case, ProMPs, to changes in the environment. Our learning system is initialized with demonstrations and potentially with trajectories based on prior knowledge about the task at hand. Gaussian Process (GP) regression is used to infer ProMPs given variables describing the current state of the environment. Pearson-Correlation-Based Relevance Weighted Policy Optimization (PRO) optimizes the inferences made by GP regression, updating the dataset of environment configurations and corresponding ProMPs, gradually improving the quality of the inferences.

To adapt ProMPs on the fly to changes in the environment, our learning system must be able to compute these ProMPs quickly. To deal with this challenge, we propose using Gaussian Process (GP) regression to map variables describing the environment to mean vector ***μ*_*w*_** and covariance matrix **Σ_*w*_** of a ProMP. Our learning system is trained according to the following steps: (1) Initialization with demonstrations and prior knowledge; (2) Given a random state of the environment, infer a ProMP using GP regression; (3) PRO optimizes upon the inferred ProMP; (4) Update dataset of environment states and corresponding ProMPs with the solution provided by PRO. Steps 2–4 are repeated several times until the learning system is able to solve a given task for a range of possible configurations of the environment. For a task in which a robot needs to move from a start position to an end position while avoiding an obstacle, for example, a suitable initialization based on prior knowledge can be a distribution of trajectories with mean going directly from the start position to the end position and a certain amount of noise for exploration by PRO. Figure 5 depicts our proposed architecture.

The vector of variables describing the current state of the environment is denoted by ***e***. The elements of this vector can be for example obstacle positions, via points, target positions, etc.

The user is asked to initialize our learning system by providing multiple demonstrations for each environment configuration ***e***_*m*_ in the set {***e***_1_, ⋯ , ***e***_*M*_} containing *M* different configurations. Based on these demonstrations, the variables *μ*_*w*_*n, m*__ and $\sigma_{w_{n,m}}^{2}$ are computed through Maximum Likelihood Estimation (MLE), where *n* ∈ ℕ, *m* ∈ ℕ, 1 ≤ *n* ≤ *N*, 1 ≤ *m* ≤ *M*. The variables *μ*_*w*_*n, m*__ and $\sigma_{w_{n,m}}^{2}$ are the mean and the variance, respectively, of weight *w*_*n*_ based on the demonstrations for environment configuration ***e***_*m*_. The set of demonstrations can be augmented for additional environment configurations by trajectories based on prior knowledge, as previously mentioned. In this case, the trajectories based on prior knowledge are treated just as the demonstrations directly provided by the user.

Given *μ*_*w*_*n, m*__ and $\sigma_{w_{n,m}}^{2},\forall m,1\leq m\leq M$, the variables *μ*_*w*_*n*__ and $\sigma_{w_{n}}^{2}$ are computed. These variables represent the average mean $\mu_{w_{n}}=\frac{1}{M}\overset{M}{\underset{m=1}{\sum }}\mu_{w_{n,m}}$ and the average variance $\sigma_{w_{n}}^{2}=\frac{1}{M}\overset{M}{\underset{m=1}{\sum }}\sigma_{w_{n,m}}^{2}$ for each weight *w*_*n*_.

A new environment ***e***_new_ is sampled at random from a set containing both the environments ***e***_1_, ⋯ , ***e***_*M*_ for which there were demonstrations as well as environments for which there were no demonstrations. Gaussian Process (GP) regression is used to infer the trajectory parameters *w*_*n*, new_ for the new configuration ***e***_new_, given the demonstrations. There is one Gaussian Process (GP) for each parameter *w*_*n*_. The GPs use the squared exponential kernel $k\left(e_{i},e_{j}\right)=\exp\left(-\alpha {\left(e_{i}-e_{j}\right)}^{⊤}\left(e_{i}-e_{j}\right)\right),\alpha \in \mathbb{R},\alpha >0$. The variables ***e***_*i*_ and ***e***_*j*_ represent any two arbitrary environment configurations. The posterior $p\left(w_{n,\text{new}}|w_{n,1:M}\right)=N\left(\mu_{w_{n,\text{new}}},\sigma_{w_{n,\text{new}}}^{2}\right)$ is a Gaussian with

(6) $$\begin{array}{l} \mu_{w_{n,\text{new}}}=\mu_{w_{n}}+K_{\text{new},1:M}{\left(K_{1:M,1:M}+\Sigma_{w_{n}}\right)}^{-1}\\ \left(\mu_{w_{n,1:M}}-\mu_{w_{n}}\right), \end{array}$$

(7) $$\begin{array}{l} \sigma_{w_{n,\text{new}}}^{2}=K_{\text{new},\text{new}}+\sigma_{w_{n}}^{2}-K_{\text{new},1:M}{\left(K_{1:M,1:M}+\Sigma_{w_{n}}\right)}^{-1}\\ \;K_{1:M,\text{new}}, \end{array}$$

where $\mu_{w_{n}}={\left[\mu_{w_{n}},\cdots \;,\mu_{w_{n}}\right]}^{⊤}$ is a column vector with μ_*w*_*n*__ repeated *M* times, $\mu_{w_{n,1:M}}={\left[\mu_{w_{n,1}},\cdots \;,\mu_{w_{n,M}}\right]}^{⊤}$, the covariance matrix of each GP is

(8) $$\begin{array}{l} K=\left(\begin{array}{cc} K_{\text{new},\text{new}} & K_{\text{new},1:M}\\ K_{1:M,\text{new}} & K_{1:M,1:M} \end{array}\right) \end{array}$$

and

(9) $$\begin{array}{l} \Sigma_{w_{n}}=\text{diag}\left(\sigma_{w_{n,1}}^{2},\cdots \;,\sigma_{w_{n,M}}^{2}\right). \end{array}$$

The covariance matrix ***K*** comprises four blocks. ***K***_new, new_ is here just the scalar *k*(***e***_new_, ***e***_new_). ***K***_new, 1:*M*_ is a row vector with elements *k*(***e***_new_, ***e***_*j*_), *j* ∈ ℕ, 1 ≤ *j* ≤ *M*. ***K***_1:*M*, new_ is a column vector with elements *k*(***e***_*i*_, ***e***_new_), *i* ∈ ℕ, 1 ≤ *i* ≤ *M*. Finally, ***K***_1:*M*, 1:*M*_ is a matrix with elements *k*(*i, j*).

After computation of the posterior distribution given by *μ*_*w*_*n*, new__ (Equation 6) and $\sigma_{w_{n,\text{new}}}^{2}$ (Equation 7), PRO optimizes upon this distribution as described in section 3. The dataset of known environments and corresponding trajectory distributions is updated with ***e***_new_, *μ*_*w*_*n*, new__ and $\sigma_{w_{n,\text{new}}}^{2}$, ∀*n*, 1 ≤ *n* ≤ *N*. Subsequently, this entire process is repeated for another ***e***_new_. After several iterations, as it will be shown in the experimental section, the learning system is able to generate successful distributions of trajectories for a pre-defined range of environment configurations.

## 5. Experiments

Four experiments demonstrate the efficacy of our proposed framework. The first experiment demonstrates that PRO can be applied to assist the teleoperation of an object in a static virtual environment. PRO achieves that by optimizing a probability distribution of trajectories based on failed attempts by the user. Depending on our reward functions, initial attempts of the user can be optimized for example to avoid obstacles, achieve a target position with higher accuracy, produce smoother and more efficient movements, etc. The next two experiments demonstrate how PRO in combination with Gaussian Process (GP) regression can tackle motion planning problems in dynamic environments. Finally, the fourth experiment demonstrates that our full framework can help users teleoperate a real robot arm in dynamic environments. Please see the Supplementary Video 1.

### 5.1. Assisted Teleoperation of a Virtual Object

In this experiment, the user manipulates the Haption Virtuose 6D to move a beam in a virtual environment (see Figure 1). This experiment can be seen as a teleoperation task, where the haptic device is the master and the beam is the slave. The goal of the user is to move the beam from a start position and orientation to an end position and orientation through the window without hitting the wall. This task is hard for humans in part due to the difficulty in visually estimating the 3D position and the orientation of the beam.

First, the user tries ten times to perform the task without force feedback. A distribution of trajectories based on the trials of the user is created using a ProMP. Subsequently, PRO is used to optimize this ProMP such that sample trajectories from the optimized ProMP pass through four via points with the right beam orientations to avoid collisions with the wall.

In this experiment, the original optimization problem has been separated into two optimization problems: one taking into consideration only the Cartesian coordinates of the via points and another taking into consideration only the orientation of the beam at each via point. This separation helped PRO to find successful trajectories in this problem. The reward function for both problems is *R*_*o*_ = exp (−*β*(*o* + *β*_*l*_*l* + *β*_*j*_*j*)), with *β* = 200, *β*_*l*_ = 0.1 and $\beta_{j}=10^{5}$. The value for *β* was empirically determined by trying a few values between 1 and 300. The values for *β*_*l*_ and *β*_*j*_ were determined by trying different powers of 10. The variable *o* represents the distances to each of the four via points. The two via points closest to the window are computed given the current position of the window. The variables *l* and *j* represent the length and the average jerk magnitude of the trajectory, respectively, and are computed by using finite differences. The terms *β*_*l*_ and *β*_*j*_ are used to regulate the importance of the length and average jerk magnitude to the reward. PRO optimized the ProMP in 150 iterations with 200 trajectory samples per iteration.

**Figure 6:**
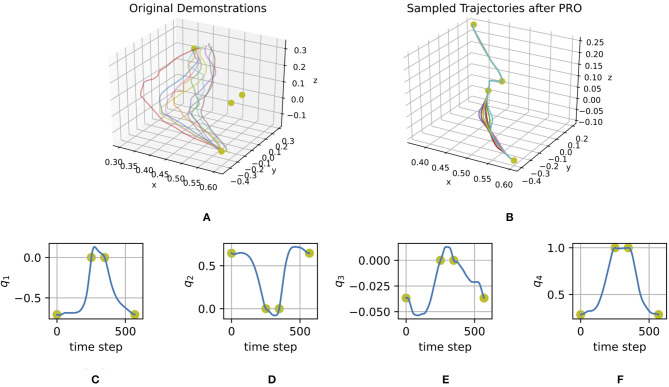
**(A)** Cartesian coordinates of demonstrated trajectories. **(B)** Cartesian coordinates of trajectories sampled from the ProMP optimized with PRO. **(C–F)** Orientations in quaternions of the mean trajectory of the ProMP optimized with PRO. The yellow dots represent via points.

Figure 6A shows the initial trials of a user to solve the task for a given scenario without the assistance of the haptic device. Figures 6B–F represents the optimized ProMP, which is used by the haptic device to guide the user with force feedback inverse proportional to the standard deviation.

### 5.2. Adaptation in Dynamic Environments—Point Particle

**Figure 7:**
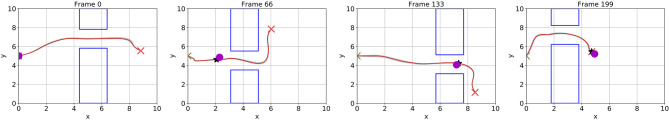
The magenta point particle has to move from the start position (green ×) to the target position (red ×) without hitting the walls (blue rectangles). The position of the hole in the wall and the target position change with time. As these positions change, our learning system computes the corresponding trajectory distributions on the fly to solve this task. The red line corresponds to the mean of the computed trajectory distribution. The light gray trajectories are samples from the computed distribution. The black star-shaped marker moves forward along the mean of the current distribution. The magenta point particle tracks the black star-shaped marker with a PD controller. This figure depicts four frames of a test case.

The problem addressed in this section is depicted in Figure 7. First, a human was presented with 30 random environments. The environments differed in ***c*** = (*x*_*c*_, *y*_*c*_), the position of the center of the hole in the wall, and in ***g*** = (*x*_*g*_, *y*_*g*_), the position of the end goal (red × in Figure 7). By using a computer mouse, the human provided three demonstrations for each environment.

The random environments $e={\left[x_{c},y_{c},x_{g},y_{g}\right]}^{⊤}$ were uniformly distributed in the range 2 ≤ *x*_*c*_ ≤ 8, 1 ≤ *y*_*c*_ ≤ 9, *x*_*c*_ + 1.5 ≤ *x*_*g*_ ≤ 10, 0 ≤ *y*_*g*_ ≤ 10. The start position ***s*** = (*x*_*s*_, *y*_*s*_) was always the same.

PRO used reward functions of the form *R*_*o*_ = exp (−*βo*), where *β* = 20 was empirically determined by observing the trajectory distributions learned by PRO for a few values of *β*: 1, 10, 20, 30, 40, and 50. In this problem, three objectives need to be minimized: the distance to the start position *o*_1_ = ||***τ***(0) − ***s***||, the minimal distance to the center of the hole in the wall $o_{2}=\min_{t}\|\tau \left(t\right)-c\|$ and the distance to the end goal *o*_3_ = ||***τ***(*T*) − ***g***||. The term ***τ***(*t*) represents the position along trajectory ***τ*** at time step *t*, ***τ***(0) is the first position and ***τ***(*T*) is the last position.

After the initialization with the demonstrations, the self-improvement loop (Figure 5) was repeated 1,000 times, each time with a new random environment. Each PRO optimization took at most 200 iterations (less if convergence was achieved sooner) and used 100 trajectory samples per iteration. The kernel of the GPs used in this problem had parameter α = 10^−3^. This value was empirically determined by trying a few different powers of 10 and observing the GP inferences. During test, our learning system can compute ProMPs on the fly, solving the task in a dynamic environment (see Figure 7).

**Figure 8:**
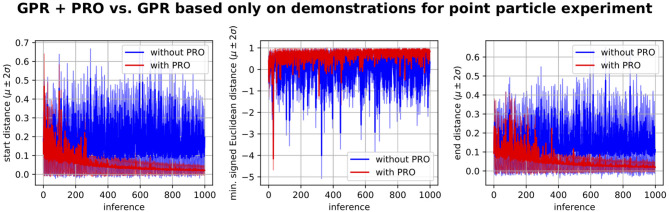
Gaussian Process regression (GPR) plus PRO vs. GPR based only on the human demonstrations without refinement by PRO. These learning curves refer to the problem depicted in Figure 7. The algorithm using GPR + PRO used GPR to compute a ProMP given a random environment, optimized the inferred ProMP by using PRO, updated its dataset of environments and corresponding ProMPs and repeated this procedure for a new random environment. The algorithm using only GPR did not optimize the GPR inferences. In this case, the dataset of environments and corresponding ProMPs was solely based on the demonstrations. Both algorithms performed 1,000 inferences of ProMPs given the same random environments. These plots show the performance measurements for the ProMPs inferred by GPR before PRO optimization for each of the 1,000 random environments. We conclude that GPR based only on the demonstrations does not generalize well to new environments as our proposed method, which uses PRO. PRO gradually improves the mapping from environments to ProMPs.

In order to verify if the performance observed during test was due to the self-optimization procedure or simply due to the human demonstrations, we have performed the comparison depicted in Figure 8. Note that by simply applying Gaussian Process Regression based on the demonstrations to infer ProMPs given a new random scenario leads to high errors with respect to the desired start and end distances as well as negative signed Euclidean distances indicating collisions with the walls. On the other hand, the self-improvement procedure involving PRO gradually leads to a better mapping from environments to ProMPs, which is evidenced by smaller errors with respect to the desired start and end positions as well as higher signed Euclidean distances to the obstacles.

### 5.3. Adaptation in Dynamic Environments—Autonomous Robot Arm

**Figure 9:**
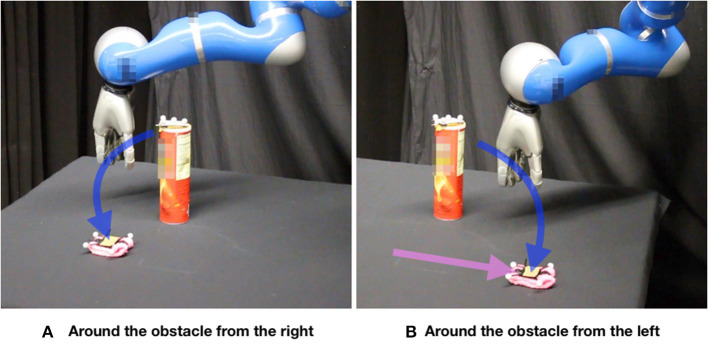
Our learning system infers a distribution of trajectories (ProMP) given the current state of the environment. An inverse-dynamics based feedback controller tracks the mean of the inferred distribution. **(A)** The robot goes around the obstacle (a cylinder) from the right side to reach the target (a pink object). **(B)** Given that the user changes the position of the target while the robot is moving, the robot switches on the fly to another ProMP, going around the obstacle from the left side.

The problem addressed in this section is depicted in Figures 2, 9. The obstacle (cylinder) and the target (pink object) are tracked by using a motion capture system (OptiTrack). First, a human provides demonstrations by moving the 7-DoF robot arm in gravity compensation mode. Demonstrations were provided for 20 different environment configurations. There were three demonstrations for each environment. The configurations were determined by arbitrarily choosing positions on the table for the obstacle and the target. The start position for the robot arm was always the same.

Each environment in this problem was represented by the vector $e={\left[x_{p},y_{p},x_{g},y_{g}\right]}^{⊤}$, where (*x*_*p*_, *y*_*p*_) was the position of the cylinder and (*x*_*g*_, *y*_*g*_) was the end goal position.

We have noticed that initializing our learning system only with the human demonstrations for 20 different situations was not enough to learn a mapping capable of dealing with any obstacle and target positions on the table. For this reason, we have decided to extend the set of demonstrations with ProMPs based on prior knowledge. These ProMPs had mean trajectory going directly from the start position to the target position irrespective of the obstacle position and the variance of each ProMP weight was the average variance for that weight based on the demonstrations. The GPs were thus initialized with ProMPs for 2,024 different environments (including environments for which human demonstrations were given). The additional 2,004 environments were generated by taking obstacle and target positions of a grid inside a range of possible positions delimited by the corners of the table and excluding configurations with the obstacle and the target too close to each other.

PRO used reward functions of the form *R*_*o*_ = exp (−*βo*), where *β* = 200 was empirically determined by observing the trajectory distributions learned by PRO for a few values of *β*: 1, 10, 20, 30, 40, 50, 100, 200 and 300. In this problem, three objectives need to be minimized: *o*_1_ = ||***τ***(0) − ***s***|| + *d*, $o_{2}=\max\left(-\min_{t}\|\tau \left(t\right)-p\|,-0.2\right)+d$ and *o*_3_ = ||***τ***(*T*) − ***g***||+*d*.

The term $d=\frac{1}{T}\sum_{t=0}^{T}\|\tau \left(t\right)-\tau_{\text{direct}}\left(t\right)\|$ is the average distance to the direct path ***τ***_direct_ from the start to the end goal. This term was added to each of the objectives to avoid large deviations from the direct path to the end goal. Apart of this term, *o*_1_ and *o*_3_ are very similar to objectives described in section 5.2. The variable ***p*** = (*x*_*p*_, *y*_*p*_) in *o*_2_ is the position of the cylinder. Minimizing *o*_2_ has the effect of avoiding the cylinder without going too far away from it because distances to the cylinder larger than 20 cm do not result in additional reward.

In the self-improvement loop (Figure 5) 2,024 inferences were made (one inference for each environment in the initialization data set). Each PRO optimization took at most 50 iterations (less if convergence was achieved sooner) and used 100 trajectory samples per iteration. The kernel of the GPs used in this problem had parameter α = 1. This value was empirically determined by trying a few different powers of 10 and observing the GP inferences. During test, the robot is able to successfully execute the reaching task even when the human moves the obstacle or the target while the robot is moving (see Figures 2, 9).

**Figure 10:**
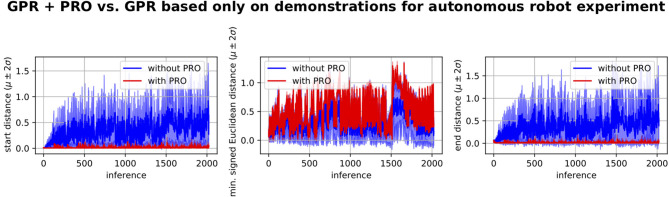
GPR + PRO vs. GPR based only on demonstrations for experiment where a 7-DoF robot arm learns to autonomously perform a reaching movement with obstacle avoidance in a dynamic environment. The performance of GPR + PRO is clearly superior. We conclude that our proposed self-optimization loop based on GPR and PRO successfully learns a mapping from environment configurations to trajectory distributions that start at the desired position, reach the target and avoid the obstacle. This learned mapping is subsequently used to produce online adaptation to changes in the environment. The performance of GPR based only on the demonstrations is relatively good for the first inferences because these inferences correspond to environments that are similar to the demonstrated ones.

As in section 5.2, we have compared the performance of our framework based on GPR and PRO with the performance of applying GPR based only on the human demonstrations. This comparison is depicted in Figure 10. The first inferences correspond to environments that are similar to the demonstrated ones. This explains why the performance of GPR based only on the demonstrations is better for the initial inferences and gradually gets worse. In any case, the performance of GPR with PRO is clearly superior to the performance of GPR only based on the demonstrations.

### 5.4. Teleoperation of a Robot Arm in a Dynamic Environment

Finally, we have used our framework to assist a human in the teleoperation of a 7-DoF robot arm. The parameters describing the environment as well as the reward functions used in this experiment are identical to the ones described in section 5.3.

**Figure 11:**
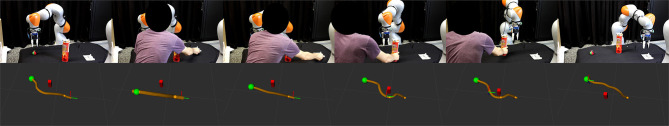
A 7-DoF robot arm is teleoperated by a user manipulating a haptic device while another human moves the objects in the slave site. The master site is depicted in the left image of Figure 1. **(Top)** Different configurations of the slave site. The objective of the operator is to move the end effector of the robot from a start position (colorful ball) to the target position (white cardboard) while avoiding the obstacle (cylinder). **(Bottom)** Corresponding virtual guides (orange tubes), virtual representation of the end effector (coordinate system), virtual representation of the obstacle (red cuboid) and virtual representation of the target (green sphere). The virtual guide adapts online to the changes in the environment.

In this experiment, a human manipulated the haptic device Haption Virtuose 6D (see the left image of Figure 1) to move a 7-DoF robot arm from a start position to a target while avoiding an obstacle. Another human changed the positions in the slave site during the teleoperation. Our framework computed online distributions of trajectories that served as virtual guides for the user. The force feedback provided to the user by the haptic device was inversely proportional to the standard deviation along the trajectory distribution. This force feedback pulled the user to the mean trajectory and allowed for free movement back and forth along the mean trajectory. Figure 11 shows a sequence of environment configurations and the corresponding virtual representation providing a visualization of the inferred virtual guides.

## 6. Conclusion and Future Work

This paper presented a new algorithm to optimize trajectory distributions, PRO. In this algorithm, the concept of Pearson correlation is used to determine the relevance of each trajectory parameter to each optimization objective. Moreover, a framework which uses PRO and GP regression has been presented, which is able to compute trajectory distributions on the fly to solve tasks in dynamic environments. PRO can be used to optimize upon suboptimal demonstrated trajectories. Our full framework is able to solve planning problems in dynamic environments in an experiment involving a point particle and in a real robot experiment with a 7-DoF robot arm. In addition, applications to the assisted teleoperation of an object in a static virtual environment as well as of a 7-DoF robot arm in a dynamic environment have been demonstrated.

In the future, we will investigate other reward functions for 6D assisted teleoperation tasks to avoid the necessity of intermediate via points and the separation in two optimization problems.

In this work, we have used Gaussian Processes with a fixed covariance function instead of addressing the model selection problem. In our case, it is a difficult problem because we do not have a supervised learning setup. The ProMPs corresponding to each new environment configuration are output by PRO, which itself gets initialized by the inferences produced by Gaussian Process Regression. As future work, it would be thus interesting to investigate to which extent model selection can be helpful in our setup.

A limitation of PRO is that it learns ProMPs with diagonal covariance matrices instead of full covariance matrices. In the problem depicted in Figure 3, if the red line had a certain slope, the solution of PRO would converge to a dot just as RWR, instead of preserving the original variance along the line. In the future, we intend to investigate the practical implications of this limitation and look for ways to learn a full covariance matrix while applying the concept of relevance functions.

## Data Availability Statement

The datasets generated for this study are available on request to the corresponding author.

## Author Contributions

ME contributed to this work by writing the manuscript, developing the proposed methods, coding, planning, preparing, and executing the experiments. GM, MT, and JP contributed to the writing of the manuscript. DK contributed to the experiments where a 7-DoF robot arm moves autonomously and adapts online to changes in the environment. OA contributed to the experiments where a 7-DoF robot arm is teleoperated and the force feedback provided to the user by the haptic device adapts online to changes in the environment. ZK contributed to the code of PRO for static scenarios and to the experiments on assisted teleoperation of an object in a virtual environment.

### Conflict of Interest

The authors declare that the research was conducted in the absence of any commercial or financial relationships that could be construed as a potential conflict of interest.

## Supplementary Material

Supplementary Video 1 Experiments on assisted teleoperation and path planning.
